# Tetra-μ-benzoato-κ^8^ *O*:*O*′-bis­[(benzoic acid-κ*O*)nickel(II)]

**DOI:** 10.1107/S1600536809044766

**Published:** 2009-10-31

**Authors:** Ji-Hua Deng, Yan-Ping Yi, Zhi-Xing Xiong, Lin Yuan, Guang-Quan Mei

**Affiliations:** a Key Laboratory of Jiangxi University for Applied Chemistry and Chemical Biology, College of Chemistry and Bio-engineering, Yichun University, Yichun 336000, People’s Republic of China

## Abstract

The title compound, [Ni_2_(C_7_H_5_O_2_)_4_(C_7_H_6_O_2_)_2_], is composed of two Ni^II^ ions, four bridging benzoate anions and two η^1^-benzoic acid mol­ecules. The [Ni_2_(PhCOO)_4_] unit adopts a typical paddle-wheel conformation. The center between the two Ni^II^ atoms represents a crystallographic center of inversion. In addition, each Ni^II^ ion also coordinates to one O atom from a benzoic acid mol­ecule. The crystal packing is realised by inter­molecular hydrogen-bonding inter­actions and π–π stacking inter­actions, with a centroid–centroid distance of 3.921 (1) Å.

## Related literature

For related benzoate complexes, see: Cotton *et al.* (2005 ▶, 1987 ▶, 1988 ▶); Bellitto *et al.* (1985 ▶); Figuerola *et al.* (2007 ▶); Gavrilenko *et al.* (2008 ▶); Shi *et al.* (2004 ▶); Zheng *et al.* (2004 ▶); Zhong *et al.* (2007 ▶, 2008 ▶).
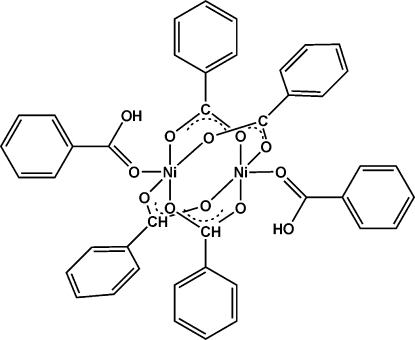


## Experimental

### 

#### Crystal data

[Ni_2_(C_7_H_5_O_2_)_4_(C_7_H_6_O_2_)_2_]
*M* *_r_* = 846.10
Monoclinic, 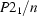

*a* = 10.7685 (8) Å
*b* = 11.7173 (7) Å
*c* = 15.258 (1) Å
β = 91.354 (3)°
*V* = 1924.7 (2) Å^3^
*Z* = 2
Mo *K*α radiation
μ = 1.04 mm^−1^
*T* = 273 K
0.28 × 0.26 × 0.20 mm

#### Data collection

Bruker SMART CCD area-detector diffractometer
Absorption correction: multi-scan (*SADABS*; Sheldrick, 2008 ▶) *T*_min_ = 0.759, *T*_max_ = 0.819
17534 measured reflections
4639 independent reflections
3789 reflections with *I* > 2σ(*I*)
*R*_int_ = 0.022

#### Refinement

*R*[*F* ^2^ > 2σ(*F* ^2^)] = 0.027
*wR*(*F* ^2^) = 0.055
*S* = 0.99
4639 reflections
254 parameters
H-atom parameters constrained
Δρ_max_ = 0.22 e Å^−3^
Δρ_min_ = −0.29 e Å^−3^

### 

Data collection: *SMART* (Bruker, 2004 ▶); cell refinement: *SAINT* (Bruker, 2004 ▶); data reduction: *SAINT*; program(s) used to solve structure: *SHELXS97* (Sheldrick, 2008 ▶); program(s) used to refine structure: *SHELXL97* (Sheldrick, 2008 ▶); molecular graphics: *SHELXTL* (Sheldrick, 2008 ▶); software used to prepare material for publication: *SHELXTL*.

## Supplementary Material

Crystal structure: contains datablocks I, global. DOI: 10.1107/S1600536809044766/im2153sup1.cif

Structure factors: contains datablocks I. DOI: 10.1107/S1600536809044766/im2153Isup2.hkl

Additional supplementary materials:  crystallographic information; 3D view; checkCIF report

## Figures and Tables

**Table 1:** Hydrogen-bond geometry (Å, °)

*D*—H⋯*A*	*D*—H	H⋯*A*	*D*⋯*A*	*D*—H⋯*A*
O6—H6⋯O3^i^	0.82	1.81	2.626 (2)	170

Symmetry code: (i) 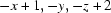.

**Fig. 1.**
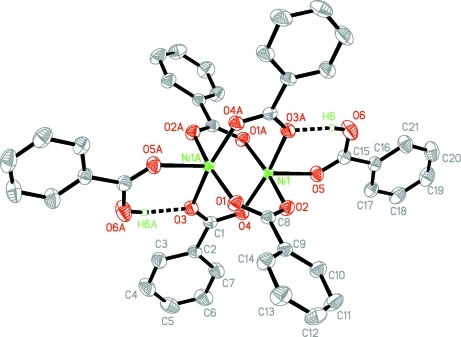
The structure of (I), showing 30% probability displacement ellipsoids and the atom-labeling scheme. [Symmetry code: (i) -x+1, -y, -z+2.]

## supplementary crystallographic information

### Comment

Benzoic acid is the most simple aromatic carboxyl compound with well-known antibacterial activity. Over the past years, many metal complexes based on benzoic acid or benzoate ligands have been synthesized and characterized (Figuerola *et al.*, 2007; Gavrilenko *et al.*, 2008; Shi *et al.*, 2004; Zheng *et al.*, 2004). We also reported the Co(II) and Cd(II) complexes with benzoate and 2-aminopyridine ligands (Zhong *et al.*, 2007; Zhong *et al.*, 2008). As a continuation of this work, we herein report the synthesis and crystal structure of a nickel (II) complex exhibiting benzoate as well as benzoic acid ligands.

The title compound (I) is a typical paddle-wheel complex that have previously been observed (Bellitto *et al.*, 1985; Cotton *et al.*, 1987; Cotton *et al.*, 1988). Two Ni^II^ ions are bridged by four benzoate anions ligand using a µ-COO^-^ coordination mode. Each Ni^II^ also coordinates to one oxygen atom from one benzoic acid molecule in the axial position (Fig. 1). The center between the two nickel atoms represents a crystallographic center of inversion. The Ni—O bond lengths, ranging from 1.945 (1) Å to 2.193 (1) Å, are in the normal value range.The almost equivalent bond distances of O1—C8 and O2—C8 (1.260 (2) Å and 1.258 (2) Å) in one benzoate ligand and O3—C1 and O4—C1 (1.270 (2) Å and 1.255 (2) Å) in the other reflect the expected delocalization in the carboxylate unit. On the other hand bond distances of O5—C15 and O6—C15 (1.213 (2) Å and 1.317 (2) Å) in the axial benzoic acid ligands prove that it is not deprotonated and accordingly there is one single and one double bond. Albeit the short Ni···Ni distance of 2.6062 (4) Å there is no bonding interaction between both metal centers due to the d^8^ electron configuration of Ni^2+^ leading to an overall bond order of zero (Cotton *et al.*, 2005)

The carboxyl group of the benzoic acid ligand (O6) acts as an intramolecular hydrogen bond donor site towards another oxygen atom of one of the bridging benzoate anions (O3).  In addition, intermolecular contacts in terms of π–π stacking interactions with centroid centroid distances of 3.921 (1) Å are observed between the phenyl groups.

### Experimental

All reagents are commercially available and were used without further purification. A mixture of Ni(NO_3_)_2_ × 4 H_2_O (0.5 mmol), sodium benzoate (1 mmol) and 2,2'-bipyridine (0.5 mmol) was dissolved in 10 ml water/methanol (1/1). After stirring for 30 min, the mixture was placed in a 20 ml Teflon-lined reactor and heated to 110 °C in an oven for 7 days. The resulting solution was filtered and the filtrate was allowed to stay at room temperature. Well shaped blue crystals suitable for X-rays diffraction were obtained after one week. Yield: 65.8% based on sodium benzoate.

### Refinement

All H atoms were placed geometrically and were refined using a riding model with C—H and O—H distances of 0.93 Å and 0.82 Å and *U*_iso_(H) = 1.2*U*_eq_(C), *U*_iso_(H) = 1.5*U*_eq_(O).

### Crystal data

**Table:**

[Ni_2_(C_7_H_5_O_2_)_4_(C_7_H_6_O_2_)_2_]	*F*(000) = 872
*M**_r_* = 846.10	*D*_x_ = 1.460 Mg m^−^^3^
Monoclinic, *P*2_1_/*n*	Mo *K*α radiation, λ = 0.71073 Å
Hall symbol: -P 2yn	Cell parameters from 7941 reflections
*a* = 10.7685 (8) Å	θ = 2.3–28.0°
*b* = 11.7173 (7) Å	µ = 1.04 mm^−^^1^
*c* = 15.258 (1) Å	*T* = 273 K
β = 91.354 (3)°	Block, blue
*V* = 1924.7 (2) Å^3^	0.28 × 0.26 × 0.20 mm
*Z* = 2	

### Data collection

**Table:**

Bruker SMART CCD area-detector diffractometer	4639 independent reflections
Radiation source: fine-focus sealed tube	3789 reflections with *I* > 2σ(*I*)
graphite	*R*_int_ = 0.022
φ and ω scans	θ_max_ = 28.1°, θ_min_ = 2.2°
Absorption correction: multi-scan (*SADABS*; Sheldrick, 2008)	*h* = −14→12
*T*_min_ = 0.759, *T*_max_ = 0.819	*k* = −15→14
17534 measured reflections	*l* = −18→20

### Refinement

**Table:**

Refinement on *F*^2^	Primary atom site location: structure-invariant direct methods
Least-squares matrix: full	Secondary atom site location: difference Fourier map
*R*[*F*^2^ > 2σ(*F*^2^)] = 0.027	Hydrogen site location: inferred from neighbouring sites
*wR*(*F*^2^) = 0.055	H-atom parameters constrained
*S* = 0.99	*w* = 1/[σ^2^(*F*_o_^2^) + 1.1987*P*] where *P* = (*F*_o_^2^ + 2*F*_c_^2^)/3
4639 reflections	(Δ/σ)_max_ = 0.002
254 parameters	Δρ_max_ = 0.22 e Å^−^^3^
0 restraints	Δρ_min_ = −0.29 e Å^−^^3^

### Special details

**Table:**

Geometry. All e.s.d.'s (except the e.s.d. in the dihedral angle between two l.s. planes) are estimated using the full covariance matrix. The cell e.s.d.'s are taken into account individually in the estimation of e.s.d.'s in distances, angles and torsion angles; correlations between e.s.d.'s in cell parameters are only used when they are defined by crystal symmetry. An approximate (isotropic) treatment of cell e.s.d.'s is used for estimating e.s.d.'s involving l.s. planes.
Refinement. Refinement of *F*^2^ against ALL reflections. The weighted *R*-factor *wR* and goodness of fit *S* are based on *F*^2^, conventional *R*-factors *R* are based on *F*, with *F* set to zero for negative *F*^2^. The threshold expression of *F*^2^ > σ(*F*^2^) is used only for calculating *R*-factors(gt) *etc*. and is not relevant to the choice of reflections for refinement. *R*-factors based on *F*^2^ are statistically about twice as large as those based on *F*, and *R*- factors based on ALL data will be even larger.

### Fractional atomic coordinates and isotropic or equivalent isotropic displacement parameters (Å^2^)

**Table:**

	*x*	*y*	*z*	*U*_iso_*/*U*_eq_	
Ni1	0.517820 (18)	0.106516 (17)	0.979210 (13)	0.03259 (6)	
O1	0.42315 (11)	−0.11994 (11)	0.89928 (7)	0.0471 (3)	
O2	0.45485 (12)	0.06289 (11)	0.86345 (8)	0.0499 (3)	
O3	0.31459 (11)	−0.05001 (10)	1.05426 (8)	0.0473 (3)	
O4	0.34725 (11)	0.13053 (10)	1.01561 (8)	0.0483 (3)	
O5	0.56860 (12)	0.28015 (11)	0.94023 (9)	0.0528 (3)	
O6	0.75910 (14)	0.25119 (13)	0.89184 (13)	0.0800 (5)	
H6	0.7451	0.1858	0.9080	0.120*	
C1	0.27981 (15)	0.05301 (15)	1.04542 (10)	0.0397 (4)	
C2	0.15133 (15)	0.08413 (16)	1.07061 (11)	0.0428 (4)	
C3	0.07758 (19)	0.0110 (2)	1.11745 (14)	0.0656 (6)	
H3	0.1080	−0.0598	1.1353	0.079*	
C4	−0.0418 (2)	0.0433 (2)	1.13776 (17)	0.0817 (7)	
H4	−0.0910	−0.0053	1.1704	0.098*	
C5	−0.0880 (2)	0.1463 (2)	1.11020 (17)	0.0768 (7)	
H5	−0.1690	0.1667	1.1230	0.092*	
C6	−0.0157 (2)	0.2189 (2)	1.06420 (18)	0.0779 (7)	
H6A	−0.0472	0.2889	1.0456	0.093*	
C7	0.10426 (18)	0.18862 (18)	1.04522 (15)	0.0623 (6)	
H7	0.1540	0.2393	1.0149	0.075*	
C8	0.41868 (15)	−0.03704 (15)	0.84671 (11)	0.0402 (4)	
C9	0.36303 (15)	−0.05843 (16)	0.75772 (11)	0.0429 (4)	
C10	0.34030 (19)	0.03208 (18)	0.70105 (12)	0.0567 (5)	
H10	0.3640	0.1056	0.7170	0.068*	
C11	0.2822 (2)	0.0125 (2)	0.62069 (14)	0.0708 (6)	
H11	0.2670	0.0731	0.5826	0.085*	
C12	0.2468 (2)	−0.0961 (2)	0.59682 (14)	0.0715 (6)	
H12	0.2077	−0.1087	0.5427	0.086*	
C13	0.2689 (2)	−0.1858 (2)	0.65259 (14)	0.0710 (6)	
H13	0.2442	−0.2591	0.6365	0.085*	
C14	0.32799 (19)	−0.16738 (18)	0.73287 (12)	0.0564 (5)	
H14	0.3442	−0.2286	0.7702	0.068*	
C15	0.66085 (18)	0.31531 (16)	0.90494 (12)	0.0464 (4)	
C16	0.67474 (18)	0.43389 (16)	0.87387 (12)	0.0470 (4)	
C17	0.5774 (2)	0.50892 (17)	0.88474 (13)	0.0563 (5)	
H17	0.5048	0.4842	0.9105	0.068*	
C18	0.5882 (2)	0.62111 (19)	0.85726 (14)	0.0684 (6)	
H18	0.5231	0.6720	0.8651	0.082*	
C19	0.6951 (3)	0.6569 (2)	0.81847 (15)	0.0758 (7)	
H19	0.7021	0.7323	0.8000	0.091*	
C20	0.7914 (3)	0.5829 (2)	0.80675 (17)	0.0809 (7)	
H20	0.8631	0.6077	0.7798	0.097*	
C21	0.7825 (2)	0.47092 (19)	0.83488 (15)	0.0670 (6)	
H21	0.8484	0.4208	0.8277	0.080*	

### Atomic displacement parameters (Å^2^)

**Table:**

	*U*^11^	*U*^22^	*U*^33^	*U*^12^	*U*^13^	*U*^23^
Ni1	0.03193 (10)	0.03109 (10)	0.03470 (10)	0.00072 (9)	−0.00051 (7)	0.00127 (9)
O1	0.0553 (7)	0.0457 (7)	0.0400 (6)	−0.0009 (6)	−0.0056 (5)	−0.0001 (6)
O2	0.0593 (8)	0.0479 (7)	0.0420 (7)	−0.0071 (6)	−0.0089 (6)	0.0007 (6)
O3	0.0406 (7)	0.0409 (7)	0.0607 (8)	0.0068 (6)	0.0076 (6)	0.0041 (6)
O4	0.0381 (6)	0.0430 (7)	0.0640 (8)	0.0051 (6)	0.0062 (6)	0.0046 (6)
O5	0.0531 (8)	0.0408 (7)	0.0648 (8)	0.0010 (6)	0.0111 (6)	0.0076 (6)
O6	0.0642 (10)	0.0483 (9)	0.1289 (15)	0.0090 (8)	0.0353 (9)	0.0193 (9)
C1	0.0377 (9)	0.0440 (10)	0.0373 (9)	0.0044 (8)	−0.0017 (7)	−0.0025 (7)
C2	0.0347 (9)	0.0493 (11)	0.0443 (9)	0.0041 (8)	0.0010 (7)	−0.0063 (8)
C3	0.0504 (12)	0.0710 (15)	0.0759 (15)	0.0086 (11)	0.0157 (10)	0.0131 (12)
C4	0.0524 (14)	0.094 (2)	0.1000 (19)	0.0019 (13)	0.0287 (13)	0.0100 (16)
C5	0.0427 (12)	0.0887 (19)	0.0995 (19)	0.0124 (12)	0.0143 (12)	−0.0141 (15)
C6	0.0511 (13)	0.0690 (16)	0.114 (2)	0.0214 (12)	0.0130 (13)	0.0013 (14)
C7	0.0466 (11)	0.0550 (13)	0.0860 (15)	0.0113 (10)	0.0142 (10)	0.0041 (11)
C8	0.0348 (9)	0.0466 (10)	0.0393 (9)	0.0026 (8)	0.0013 (7)	−0.0023 (8)
C9	0.0380 (9)	0.0526 (11)	0.0381 (9)	−0.0003 (8)	−0.0004 (7)	−0.0031 (8)
C10	0.0632 (13)	0.0571 (13)	0.0493 (11)	−0.0062 (10)	−0.0107 (9)	0.0030 (9)
C11	0.0832 (16)	0.0773 (16)	0.0508 (12)	0.0016 (13)	−0.0206 (11)	0.0083 (11)
C12	0.0783 (16)	0.0869 (18)	0.0483 (12)	0.0023 (14)	−0.0214 (11)	−0.0103 (12)
C13	0.0864 (17)	0.0681 (15)	0.0578 (13)	−0.0087 (13)	−0.0151 (12)	−0.0166 (11)
C14	0.0677 (13)	0.0549 (12)	0.0463 (10)	−0.0010 (10)	−0.0069 (9)	−0.0046 (9)
C15	0.0518 (11)	0.0414 (10)	0.0460 (10)	0.0004 (9)	0.0013 (8)	−0.0009 (8)
C16	0.0575 (11)	0.0395 (10)	0.0441 (10)	−0.0042 (9)	−0.0002 (8)	−0.0006 (8)
C17	0.0650 (13)	0.0499 (12)	0.0539 (12)	0.0032 (10)	−0.0006 (10)	0.0029 (9)
C18	0.0915 (17)	0.0472 (13)	0.0662 (14)	0.0111 (12)	−0.0076 (12)	0.0016 (10)
C19	0.112 (2)	0.0447 (13)	0.0706 (15)	−0.0124 (14)	−0.0019 (14)	0.0078 (11)
C20	0.0904 (19)	0.0598 (16)	0.0932 (18)	−0.0205 (14)	0.0171 (15)	0.0114 (13)
C21	0.0697 (15)	0.0512 (13)	0.0808 (15)	−0.0051 (11)	0.0141 (12)	0.0040 (11)

### Geometric parameters (Å, °)

**Table:**

Ni1—O2	1.9452 (12)	C7—H7	0.9300
Ni1—O4	1.9519 (12)	C8—C9	1.492 (2)
Ni1—O1^i^	1.9520 (11)	C9—C14	1.382 (3)
Ni1—O3^i^	1.9999 (12)	C9—C10	1.386 (3)
Ni1—O5	2.1926 (13)	C10—C11	1.382 (3)
Ni1—Ni1^i^	2.6062 (4)	C10—H10	0.9300
O1—C8	1.260 (2)	C11—C12	1.375 (3)
O1—Ni1^i^	1.9520 (11)	C11—H11	0.9300
O2—C8	1.258 (2)	C12—C13	1.370 (3)
O3—C1	1.270 (2)	C12—H12	0.9300
O3—Ni1^i^	1.9999 (12)	C13—C14	1.384 (3)
O4—C1	1.255 (2)	C13—H13	0.9300
O5—C15	1.213 (2)	C14—H14	0.9300
O6—C15	1.317 (2)	C15—C16	1.477 (3)
O6—H6	0.8200	C16—C17	1.381 (3)
C1—C2	1.490 (2)	C16—C21	1.386 (3)
C2—C3	1.379 (3)	C17—C18	1.385 (3)
C2—C7	1.377 (3)	C17—H17	0.9300
C3—C4	1.382 (3)	C18—C19	1.373 (3)
C3—H3	0.9300	C18—H18	0.9300
C4—C5	1.368 (3)	C19—C20	1.367 (3)
C4—H4	0.9300	C19—H19	0.9300
C5—C6	1.359 (3)	C20—C21	1.384 (3)
C5—H5	0.9300	C20—H20	0.9300
C6—C7	1.377 (3)	C21—H21	0.9300
C6—H6A	0.9300		
			
O2—Ni1—O4	89.20 (5)	O2—C8—O1	125.54 (16)
O2—Ni1—O1^i^	169.19 (5)	O2—C8—C9	117.17 (16)
O4—Ni1—O1^i^	90.32 (5)	O1—C8—C9	117.27 (16)
O2—Ni1—O3^i^	88.77 (5)	C14—C9—C10	119.50 (17)
O4—Ni1—O3^i^	168.91 (5)	C14—C9—C8	120.40 (17)
O1^i^—Ni1—O3^i^	89.64 (5)	C10—C9—C8	120.01 (17)
O2—Ni1—O5	94.67 (5)	C11—C10—C9	119.7 (2)
O4—Ni1—O5	100.71 (5)	C11—C10—H10	120.1
O1^i^—Ni1—O5	96.03 (5)	C9—C10—H10	120.1
O3^i^—Ni1—O5	90.32 (5)	C12—C11—C10	120.4 (2)
O2—Ni1—Ni1^i^	85.36 (4)	C12—C11—H11	119.8
O4—Ni1—Ni1^i^	85.63 (4)	C10—C11—H11	119.8
O1^i^—Ni1—Ni1^i^	83.83 (4)	C13—C12—C11	120.16 (19)
O3^i^—Ni1—Ni1^i^	83.34 (4)	C13—C12—H12	119.9
O5—Ni1—Ni1^i^	173.66 (4)	C11—C12—H12	119.9
C8—O1—Ni1^i^	123.31 (11)	C12—C13—C14	120.0 (2)
C8—O2—Ni1	121.90 (11)	C12—C13—H13	120.0
C1—O3—Ni1^i^	123.58 (11)	C14—C13—H13	120.0
C1—O4—Ni1	123.73 (11)	C13—C14—C9	120.3 (2)
C15—O5—Ni1	130.36 (12)	C13—C14—H14	119.9
C15—O6—H6	109.5	C9—C14—H14	119.9
O4—C1—O3	123.69 (15)	O5—C15—O6	122.89 (17)
O4—C1—C2	117.72 (15)	O5—C15—C16	123.51 (18)
O3—C1—C2	118.58 (16)	O6—C15—C16	113.59 (17)
C3—C2—C7	119.05 (17)	C17—C16—C21	119.83 (19)
C3—C2—C1	122.08 (17)	C17—C16—C15	118.53 (18)
C7—C2—C1	118.87 (17)	C21—C16—C15	121.64 (18)
C2—C3—C4	119.7 (2)	C16—C17—C18	119.9 (2)
C2—C3—H3	120.1	C16—C17—H17	120.0
C4—C3—H3	120.1	C18—C17—H17	120.0
C5—C4—C3	120.4 (2)	C19—C18—C17	119.8 (2)
C5—C4—H4	119.8	C19—C18—H18	120.1
C3—C4—H4	119.8	C17—C18—H18	120.1
C6—C5—C4	120.1 (2)	C18—C19—C20	120.6 (2)
C6—C5—H5	119.9	C18—C19—H19	119.7
C4—C5—H5	119.9	C20—C19—H19	119.7
C5—C6—C7	119.9 (2)	C19—C20—C21	120.2 (2)
C5—C6—H6A	120.0	C19—C20—H20	119.9
C7—C6—H6A	120.0	C21—C20—H20	119.9
C6—C7—C2	120.7 (2)	C20—C21—C16	119.7 (2)
C6—C7—H7	119.6	C20—C21—H21	120.2
C2—C7—H7	119.6	C16—C21—H21	120.2

Symmetry codes: (i) −*x*+1, −*y*, −*z*+2.

### Hydrogen-bond geometry (Å, °)

**Table:**

*D*—H···*A*	*D*—H	H···*A*	*D*···*A*	*D*—H···*A*
O6—H6···O3^i^	0.82	1.81	2.626 (2)	170

Symmetry codes: (i) −*x*+1, −*y*, −*z*+2.
